# Une éruption cutanée élective du dos révélatrice de lupus érythémateux systémique

**DOI:** 10.11604/pamj.2013.15.77.2784

**Published:** 2013-06-26

**Authors:** Faida Ajili, Najeh Boussetta

**Affiliations:** 1 Service de médecine interne, Hôpital militaire de Tunis, Tunisie

**Keywords:** Eruption cutanée, lupus, rash, lupus

## Images en médecine

Les manifestations cutanées au cours du lupus érythémateux systémique ont une grande valeur diagnostique. Plusieurs types de lésions sont observés. Si certaines lésions sont fortement évocatrices du diagnostic de part leur aspect et leur siège d'autres moins typiques nécessitent le recours à la biopsie pour confirmer le diagnostic. Nous rapportons l'observation d'une patiente âgée de 40 ans qui a été hospitalisée dans un tableau d'éruption cutanée avec fièvre et polyarthralgie. A l'examen, la patiente était fébrile à 38°C, elle présentait une éruption vésiculeuse, squameuse par endroit , non prurigineuses localisée uniquement au niveau du dos. Les zones photosensibles (visage, cou, mains) ont étaient épargné. La tension artérielle était à 120/70 mmHg, l'auscultation cardio-pulmonaire était normale et l'examen ostéoarticulaire avait montrait une arthrite du genou gauche. Les examens neurologique et abdominal étaient sans particularités. A la biologie, La NFS montrait une thrombopénie à 100000 éléments/mm3, la protéinurie de 24h était à 2,28g/24h, la CRP était à 40 mg/l, La VS à 50 mm première heure, la créatinine était à 160 µmol/l et l'ionogramme était normal. Une enquête étiologique a éliminé les causes infectieuses virales et bactériennes et devant ce tableau le diagnostic de lupus érythémateux systémique a été suspecté malgré le caractère atypique de l'éruption cutanée. Les AAN étaient positifs à 1/1280, les anti DNA natifs étaient positifs et la biopsie cutanée des lésions du dos à montré un dépôt d'IgM, IgG, IgA et de complément au niveau de la jonction dermoépidermique. Le diagnostic de LES été retenu et la patiente à été mise sous corticothérapie à la dose de 1mg/kg/j avec une amélioration clinique notable, une disparition quasi totale des lésions cutanées et une augmentation du taux de plaquettes permettant la réalisation de la ponction biopsie rénale qui a montré une glomérulonéphrite diffuse proliférative classe IV. Le cyclophosphamide à été alors débuté. La présentation clinique variable de l'atteinte cutanée au cours du lupus incite à analyser de façon rigoureuse toute lésion dermatologique et à s'aider au besoin de l'examen anatomopathologique pour confirmer le diagnostic.

**Figure 1:**
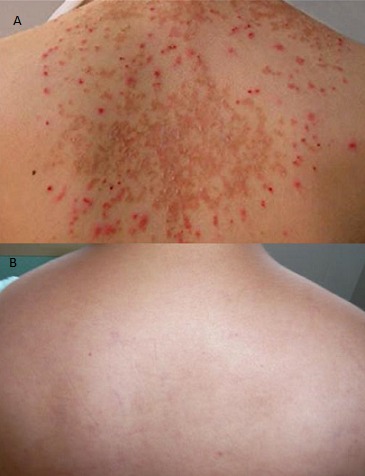
A) Eruption vésiculeuse, squameuse par endroit au niveau du dos (avant traitement). B) disparition des lésions cutanées du dos après traitement corticoïdes

